# Identification and Characterization of a Novel Genomic Island Harboring Cadmium and Arsenic Resistance Genes in *Listeria welshimeri*

**DOI:** 10.3390/biom11040560

**Published:** 2021-04-11

**Authors:** Sangmi Lee, Cameron Parsons, Yi Chen, Zahra Hanafy, Eric Brown, Sophia Kathariou

**Affiliations:** 1 Department of Food and Nutrition, Chungbuk National University, Chengju, Chungbuk 28644, Korea; 2 Department of Food, Bioprocessing and Nutrition Sciences, North Carolina State University, Raleigh, NC 27695-7624, USA; ctparson@ncsu.edu (C.P.); zehanafy@ncsu.edu (Z.H.); skathar@ncsu.edu (S.K.); 3 Division of Microbiology, Center for Food Safety and Applied Nutrition, Food and Drug Administration, College Park, MD 20740-3835, USA; Yi.Chen@fda.hhs.gov (Y.C.); Eric.Brown@fda.hhs.gov (E.B.)

**Keywords:** *Listeria welshimeri*, *Listeria* genomic island 2 (LGI2), LGI2-3, heavy metal resistance, arsenic, cadmium

## Abstract

*Listeria monocytogenes,* the bacterial foodborne pathogen responsible for the severe disease listeriosis, frequently exhibits heavy metal resistance. Concurrent resistance to cadmium and arsenic in *L. monocytogenes* is strongly associated with the 35-kb chromosomal island LGI2. LGI2 has been encountered repeatedly among *L. monocytogenes* serotype 4b hypervirulent clones but, surprisingly, not among non-pathogenic *Listeria* spp. Here we describe a novel LGI2 variant, LGI2-3, in two *L. welshimeri* strains from an urban aquatic environment. Whole genome sequence analysis revealed that the genomes were closely related except for one prophage region and confirmed a chromosomally integrated LGI2-3. It harbored a cystathionine beta-lyase gene previously only encountered in LGI2-1 of *L. monocytogenes* clonal complex 1 but was otherwise most closely related to LGI2. LGI2-3 harbored a novel *cadAC* cassette (*cadA7C7)* that, like LGI2′s *cadA4C4*, was associated with lower-level tolerance to cadmium (MIC 50 μg/mL) than other *cadAC* cassettes (MIC ≥ 140 μg/mL). CadA sequence analysis identified two amino acids that may be important for mediating different levels of cadmium tolerance. Our findings clearly demonstrated the potential for LGI2-like islands to be harbored by non-pathogenic *Listeria* spp. and generate intriguing hypotheses on the genetic diversity mediated by this island and its transfer among *Listeria* spp.

## 1. Introduction

*Listeria monocytogenes* is the only human pathogen in the genus *Listeria* and is responsible for the severe foodborne disease listeriosis, which is characterized by symptoms such as stillbirths, meningitis and septicemia, and has high hospitalization and case fatality rates [1,2,3,4]. The 13 known serotypes of *L. monocytogenes* are classified into four lineages (I to IV); the lineage II serotype 1/2a and the lineage I serotypes 1/2b and 4b contribute to the majority of human cases of listeriosis [5,6,7]. Serotype 4b is especially noteworthy for its frequent involvement in outbreaks and the presence of hypervirulent clones of clonal complexes (CCs) 1, 2, 4 and 6 [6,7,8,9].

*L. monocytogenes* is notorious for its capacity to persistently colonize food-processing environments and equipment [6,10,11]. Persistence in food-processing environments is mediated by multiple mechanisms and adaptive traits of *L. monocytogenes*, including the capacity to grow at low temperatures, biofilm formation, and resistance to disinfectants and phages [6,10,11,12].

Resistance to heavy metals, especially cadmium and arsenic, has long been recognized as a major adaptation of *L. monocytogenes* [13,14]. Resistance to cadmium has been extensively investigated, leading to the identification of a large array of *cadAC* cadmium resistance cassettes, with *cadA* and *cadC* encoding a cadmium ATPase mediating efflux of cadmium and a transcriptional regulator, respectively [15,16]. Some of these cadmium resistance determinants have also been identified in non-pathogenic *Listeria* spp., where they exhibit the potential to transfer between different *Listeria* spp., sometimes along with benzalkonium chloride resistance genes on the same mobile genetic elements [17,18,19].

Interestingly, in *L. monocytogenes* serotype 4b, cadmium resistance is often encountered together with resistance to arsenic, especially among strains of the predominant, hypervirulent clones CC1 and CC2 [16,20]. This concurrent resistance to cadmium and arsenic is mediated by *Listeria* genomic island (LGI) 2, a 35-kb chromosomal region that harbors genes mediating arsenic and cadmium resistance, DNA integration, conjugation, and pathogenicity [16,21]. LGI2 was originally identified in serotype 4b CC2 strain Scott A and harbors a cadmium resistance cassette (*cadA4C4*) that conferred resistance to lower levels of cadmium (35 μg/mL) in comparison to other *cadAC* cassettes (70 μg/mL) [21,22,23]. Further studies revealed that LGI2 was harbored by diverse strains of *L. monocytogenes*, with a predilection for the serotype 4b hypervirulent clones CC1 and CC2 [9,21,24].

LGI2 was identified in multiple genomic locations, often disrupting open reading frames and exhibiting diversity in sequence content [21]. A notably diversified variant of LGI2, designated LGI2-1, was identified in certain strains of *L. monocytogenes* CC1 [21]. LGI2-1 was characterized by the presence of a unique novel gene encoding a putative cystathionine gamma-synthase immediately upstream of arsenic resistance genes, as well as by its capacity to confer resistance to a higher concentration of cadmium than conferred by LGI2 [16,20,21]. The capacity of strains harboring LGI2-1 to tolerate higher levels of cadmium appears to be mediated by a distinct resistance cassette, *cadA5C5*, which, like *cadA4C4,* has to date been encountered only on LGI2 and LGI2-like islands integrated in the chromosome of *L. monocytogenes* [16,21]. 

LGI2 and LGI2-like islands appear to be uncommon in *L. monocytogenes* serotypes other than 4b, and arsenic resistance in serotypes 1/2a and 1/2c is frequently mediated by Tn*554* [21,24]. Even more surprisingly, LGI2 and its variants have until now appeared to be restricted to *L. monocytogenes*. Even though as discussed above several *cadAC* cadmium resistance determinants have been detected in non-pathogenic *Listeria* spp., *cadA4C4* or *cadA5C5* have not yet been reported [17,18,19]. Furthermore, arsenic resistance in these non-pathogenic spp. appears to be mediated by plasmid-borne genes, as in the *L. innocua* CLIP11262 plasmid pLI100, or by the arsenic resistance transposon Tn*554* [16,18,19,25,26].

Considering the remarkable ability of LGI2 and LGI2-like islands to detoxify both cadmium and arsenic, especially in hypervirulent clones of *L. monocytogenes*, a further understanding of the ecology and evolution of these islands would be highly valuable. In this context, the possible discovery and characterization of these islands in non-pathogenic *Listeria* spp. may help address current gaps in the emergence, dissemination and adaptive physiology roles of these intriguing mobile genetic elements. Here, our objective was to characterize the genomic basis for concurrent resistance to cadmium and arsenic that we noted in two strains of the non-pathogenic spp. *L. welshimeri* recently isolated from an urban aquatic environment. Whole genome sequence analysis was employed to determine whether these strains harbored LGI2-like elements and to compare them to those previously identified in *L. monocytogenes*. In addition, the sequence data were employed to facilitate comparisons among *cadAC* cassettes to identify features that may be associated with different cadmium tolerance levels. 

## 2. Materials and Methods 

### 2.1. Bacterial Strains and Growth Media

Strains investigated in this study (*L. welshimeri* SKWL416 and SKWL425) were isolated as described below in the course of the analysis of 66 samples from swabs of submerged rocks and other submerged objects (*n* = 33) and surrounding water (*n* = 33) in Rocky Branch Creek, a freshwater creek in an urban area in Raleigh, NC, USA. *L. welshimeri* SKWL416 was isolated on 22 January 2019 from the swab of a rock submerged in the creek in an area with normal water flow and surrounding water temperature of 4.5 °C, while *L. welshimeri* SKWL425 was isolated on 29 January 2019, from the swab of another submerged rock in fast-flowing water with a temperature of 9 °C. The strains were routinely grown at 37 °C in a tryptic soy broth supplemented with 0.6% yeast extract (Becton, Dickinson and Co., Sparks, MD, USA), and preserved in brain heart infusion (BHI) broth (Becton, Dickinson and Co.) with 20% glycerol, as described [27].

### 2.2. Isolation and Characterization of L. welshimeri

Samples (swabs and water) were placed on ice and transported to the laboratory where they were processed within 2 h. Enrichments for *Listeria* spp. were as described [27]. Briefly, for the primary enrichment the water samples were diluted 1:10 (1.25 mL in 11.25 mL) in half Fraser broth supplemented with half Fraser selective supplement (Oxoid, Hampshire, UK) and incubated at 30 °C for 24–48 h. For swabs, the swab tip was aseptically removed, placed in 11.25 mL of the same primary enrichment broth with the supplement and similarly incubated. Then, 100 µL of the primary enrichment was added to 10 mL of full Fraser broth with full Fraser selective supplement (Oxoid) and incubated at 37 °C for 48 h. Primary and secondary enrichments (20 µL) were spread-plated on modified Oxford medium (MOX; Becton, Dickinson and Co.) and incubated at 37 °C for 48 h. Colonies typical of *Listeria* spp. were purified on trypticase soy agar with sheep blood (Remel, San Diego, CA, USA) and analyzed via multiplex PCR as described [28]. Resistance to benzalkonium chloride and the heavy metals cadmium and arsenic was determined as described [29], with the modification that cadmium resistance was determined at both 35 and 70 μg/mL cadmium chloride anhydrous (Sigma, St. Louis, MO, USA), and arsenic resistance was tested at 500, 1000 and 2000 μg/mL sodium arsenite (Fluka, Buchs, Switzerland).

### 2.3. Whole Genome Sequencing (WGS) and Analysis

SKWL416 and SKWL425 were grown overnight at 37 °C in BHI broth and total genomic DNA was extracted using the DNeasy blood and tissue kit (QIAGEN, Valencia, CA, USA). Genomic DNA (1 ng) was used to construct libraries with a Nextera XT DNA library preparation kit (Illumina, San Diego, CA, USA). WGS was conducted with a NextSeq 500 sequencer with the NextSeq 500/550 high-output kit v2.5 (300 cycles, 2 × 150 bp) (Illumina) according to the manufacturer’s instructions. Raw sequence reads were quality-trimmed and de novo assembled using CLC Genomics Workbench v. 20 (QIAGEN, Aarhus, Denmark), and quality assessment of the assemblies employed QUAST v.4.6.4 [30]. The whole genome sequence data for SKWL416 and SKWL425 were deposited in the National Center for Biotechnology Information (NCBI) under accession nos. CFSAN096004 (SRR13284904) and CFSAN096006 (SRR13284912), respectively.

All software applications used default parameters. In silico *Listeria* spp. designations were assigned based on average nucleotide identity analysis performed using the pyani software package (https://github.com/widdowquinn/pyani (accessed on 18 March 2021)). Multilocus sequence typing (MLST) and identification of sequence type (ST) and CC were performed in silico with the MLST package (https://github.com/tseemann/mlst (accessed on 18 March 2021)), using the PubMLST database (https://bigsdb.pasteur.fr/listeria/listeria.html (accessed on 18 March 2021)) curated by the Institut Pasteur. The CFSAN SNP Pipeline was used to assess strain-level nucleotide differences, using default settings as previously described [31].

**Figure 1:**
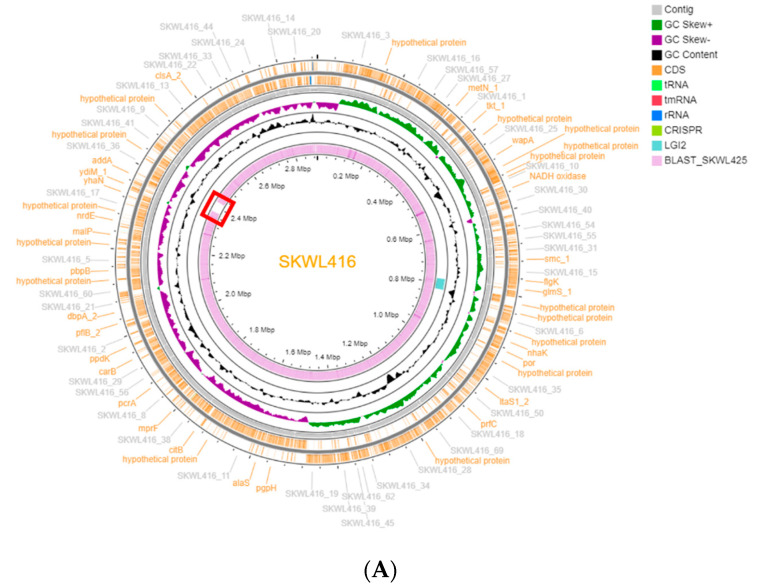

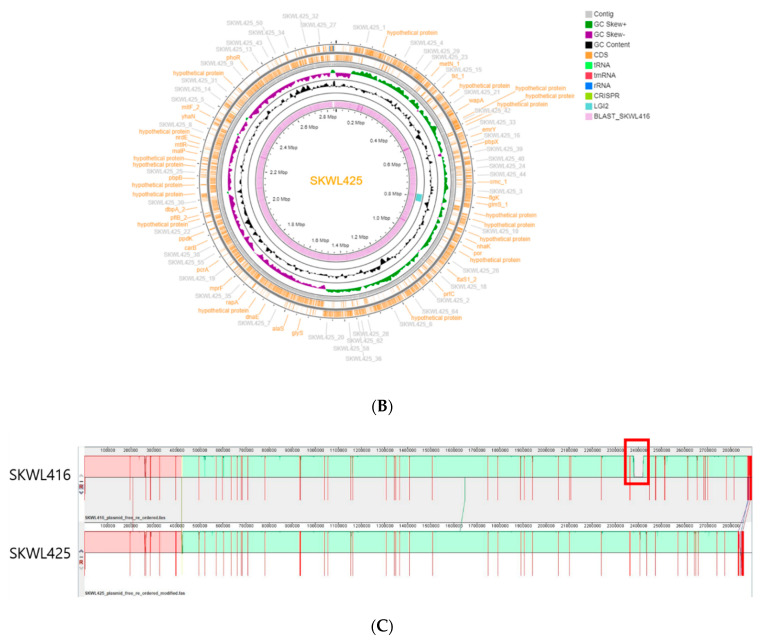
Analysis of whole genome sequence data and identification of LGI2 in *L. welshimeri* strains SKWL416 and SKWL425. The location of LGI2 and the strain-specific region are shown in the circular maps of the genomes of *L. welshimeri* SKWL416 (**A**) and SKWL425 (**B**). Circular maps were drawn with CGView Server (http://cgview.ca/ (accessed on 18 March 2021)) [35]. The contigs did not include plasmid-associated contigs identified via RFPlasmid (http://klif.uu.nl/rfplasmid/ (accessed on 18 March 2021)) [33], were re-ordered with Mauve Contig Mover [34], and were manually inspected as described in Materials and Methods. The genomes were annotated with Prokka [32], yielding genetic features that are shown in the circular maps as rectangular boxes in the two outermost circles separated by the dark gray line. Individual features can be visualized at higher resolution in the zoomable maps created using the json files of the circular maps (Supplementary Materials S1 and S2). Coding sequences (CDSs), tRNAs, tmRNAs, CRISPRs and rRNAs are marked in dark yellow, neon, red, blue and light green, respectively, and several annotations are shown in dark yellow outside the circle. Contigs are represented in the third outermost circle as light gray arrows with contig names shown in light gray outside the circle. Positive and negative GC skew indices are indicated in dark green and purple, respectively, followed by a circle depicting the deviation from the genome mean GC percent (marked as GC content in the inset). The two innermost circles indicate the location of LGI2 (light blue rectangle), which was determined by visualizing the output of BLAST2 between the mapped genome and its LGI2 region, and the BLAST2 output between *L. welshimeri* SKW416 and SKWL425 (homologous regions shown in light purple). The strain-specific region in SKWL416 is marked with a red rectangle. (**C**) *L. welshimeri* SKWL416 and SKWL425 genomes were also compared with Mauve [34]. Highly homologous regions are shown as rectangles whereas divergent regions are indicated with jagged peaks. Homologous regions are marked in the same color. The strain-specific region in SKWL416 is marked with a red rectangle.

The contigs were annotated with Prokka (version 1.14.5; included in the Docker image available at https://hub.docker.com/r/staphb/prokka (accessed on 18 March 2021)) [32]. Plasmid-borne contigs were identified using RFPlasmid (http://klif.uu.nl/rfplasmid/ (accessed on 18 March 2021)) [33] and eliminated from further analysis. The plasmid-free contigs were re-ordered with Mauve Contig Mover included in Mauve (version 20150226 build 10 (c)) [34] against the complete genome of *L. welshimeri* SLCC5334 (accession no. AM263198). Re-ordered contigs were manually inspected and visualized as a circular map with CGView Server (http://cgview.ca/ (accessed on 18 March 2021)) [35] (Figure 1A,B). To compare the SKWL416 and SKWL425 genomes, homologous regions identified via BLAST2 of the two genomes [36] were visualized on the circular map of each genome. Strain-specific regions were also identified with the alignment of the two genomes via Mauve [34] (Figure 1C). Each genome was analyzed with the web-based phage identification program PHASTER (https://phaster.ca/ (accessed on 18 March 2021)) [37]. To calculate the size and GC content of the plasmid-free re-ordered contigs, all contigs were concatenated into a single sequence, and bioawk (https://github.com/lh3/bioawk (accessed on 18 March 2021)) was used to calculate genome size and GC content.

### 2.4. Identification and Analysis of LGI2 in L. welshimeri

To detect the presence of LGI2, the contigs of SKWL416 and SKWL425 were analyzed with Genome Comparator (https://bigsdb.pasteur.fr/cgi-bin/bigsdb/bigsdb.pl?db=pubmlst_listeria_isolates&page=plugin&name=GenomeComparator (accessed on 18 March 2021)) with default parameters using the scheme of *Listeria* Genomic Islands. The Genome Comparator output indicated the presence or absence of a homolog and its allele number. The LGI2 genes in SKWL416 and SKWL425 were identified with BLAST2 between each genome and the LGI2 region in *L. monocytogenes* strain Scott A (accession no. NZ_CP023862). To identify the homologs of the LGI2 flanking genes in the *L. monocytogenes* F2365 genome, we performed BLAST2 of the flanking genes against F2365 genome (accession no. NC_002973) and visualized the output in the circular map of the F2365 genome drawn with CGView Server.

**Figure 2:**
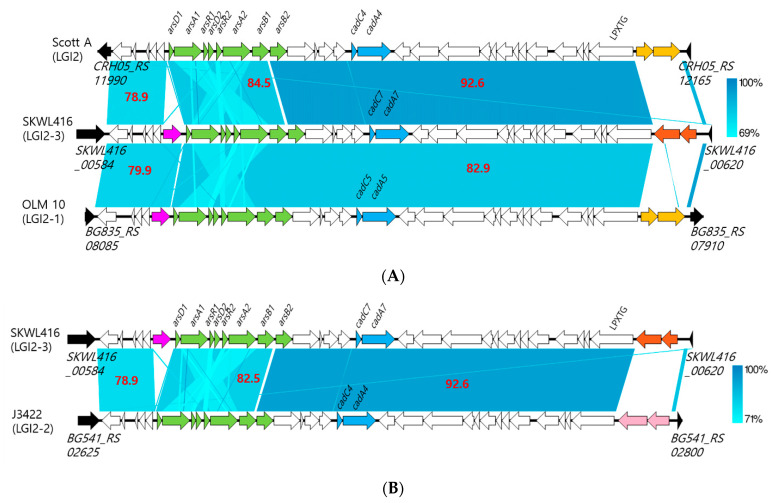
Comparison of LGI2-3 in *L. welshimeri* SKWL416 with LGI2 and other LGI2 variants. The LGI2 region (LGI2-3) in *L. welshimeri* SKWL416 was compared with the LGI2 of *L. monocytogenes* Scott A (accession no. NZ_CP023862) and LGI2-1 of *L. monocytogenes* OLM 10 (accession nos. NZ_MIMA01000001 to NZ_MIMA01000072) (**A**) and the LGI2-2 of *L. monocytogenes* J3422 (accession nos. NZ_MNCC01000001 to NZ_MNCC01000116) (**B**). BLAST2 using the blastn algorithm was implemented to identify homologous regions, which were then visualized with Easyfig as described in Materials and Methods. The shade of blue indicates the similarity of homologous regions as designated in the gradient bar. Numbers in red font indicate sequence similarity (%) of major homologous regions in pairwise comparisons. Genes are represented as arrows indicating direction of transcription. Chromosomal genes flanking LGI2 and its variants are in black. Inside LGI2 and its variants, homologous genes are shown in the same color. Genes mediating detoxification of arsenic and cadmium are marked in green and blue, respectively; cystathionine beta-lyase gene is in magenta; and those downstream of the LPXTG protein gene are signified by yellow (homologs found in LGI2 and LGI2-1), pink (LGI2-2) and orange (LGI2-3). Other genes within the islands are marked in white. Locus tags of the flanking genes are shown below the corresponding arrows and the designations of arsenic and cadmium resistance genes and the LPXTG protein gene are above the corresponding arrows.

Regions containing the LGI2 and flanking genes were extracted from the GenBank files of the SKWL416 and SKWL425 genomes, which were generated by Prokka, and the resulting GenBank files were converted to FASTA format. LGI2 regions in *L. welshimeri* strains SKWL416 and SKWL425 were compared using BLAST2 between each other and with previously identified LGI2 variants present in *L. monocytogenes* strains OLM 10 and J3422 as well as the LGI2 in Scott A [21] and these pairwise comparisons were visualized with Easyfig [38]. To retrieve the sequences of LGI2 variants from OLM 10 and J3422, all the contigs of each genome (accession nos. NZ_MIMA01000001 to NZ_MIMA01000072 for OLM 10 and NZ_MNCC01000001 to NZ_MNCC01000116 for J3422) were downloaded from NCBI in GenBank and FASTA formats and the sequences of the LGI2 variants were extracted after identifying the insertion sites (*LMOf2365_0902* homolog for OLM 10 and the intergenic region between *LMOf2365_2381* and *LMOf2365_2382* homologs for J3422) [21] with BLAST2. Genes found only in the LGI2 regions of *L. welshimeri* strains SKWL416 and SKWL425 were subjected to BLASTp and Batch-Conserved Domain Searches (https://www.ncbi.nlm.nih.gov/Structure/bwrpsb/bwrpsb.cgi (accessed on 18 March 2021)) to infer their functions and identify possible homologs [36,39]. Analysis with BLAST2 used the megablast algorithm except for the pairwise comparisons visualized in Figure 2, which used the blastn algorithm. BLAST2 (version 2.10.0+) and acquisition of the genome data from NCBI employed the Docker images provided by NCBI (available at https://hub.docker.com/r/ncbi/blast (accessed on 18 March 2021) and https://hub.docker.com/r/ncbi/edirect (accessed on 18 March 2021), respectively). Sequence manipulations were performed with custom scripts using Bash and Biopython [40]. BLASTp was conducted on the NBCI website (https://blast.ncbi.nlm.nih.gov/Blast.cgi?PROGRAM=BLASTx&PAGE_TYPE=BlastSearch&LINK_LOC=blasthome (accessed on 18 March 2021)). The size and GC content of the LGI2 regions were calculated using bioawk as indicated above after eliminating the flanking genes.

To detect any *L. welshimeri* genomes bearing the LGI2 variant identified in *L. welshimeri* strains SKWL416 and SKWL425, BLASTn was employed against *L. welshimeri* genomes in the NCBI database with SKWL416 LGI2 as query. The total number of *L. welshimeri* genomes was calculated using keywords “((Listeria welshimeri[Organism]) AND genome[Title]) AND complete[Title]” and “((Listeria welshimeri[Organism]) AND genome[Title]) AND project[Title]” in the Nucleotide database in NCBI and redundant RefSeq entities were subtracted.

### 2.5. Comparison of Cadmium Resistance Determinants

Protein sequences encoded by the following *cadA* and *cadC* genes in LGI2 variants were extracted from the GenBank files of LGI2 variants: *cadA4C4*: *CRH05_RS12085* and *CRH05_RS12080* in *L. monocytogenes* Scott A and *BG541_RS02720* and *BG541_RS02715* in *L. monocytogenes* J3422; *cadA5C5*: *BG835_RS07990* and *BG835_RS07995* in *L. monocytogenes* OLM 10; and *cadA7C7*: *SKWL416_00604* and *SKWL416_00603* in SKWL416 and *SKWL425_01225* and *SKWL425_01226* in SKWL425. To obtain the protein sequences encoded in non-LGI2-associated *cadAC* genes, we downloaded the GenBank files of the DNA sequences containing *cadAC* genes: Tn*5422*-associated *cadAC* for *cadA1C1* (accession no. L28104); contig 506 of plasmid pLM80 harbored by *L. monocytogenes* H7858 for *cadA2C2* (accession no. AADR01000058); *L. monocytogenes* EGD-e complete genome for *cadA3C3* (accession no. NC_003210); *L. seeligeri* Sr12 plasmid pLIS4 for *cadA6aC6a* (accession no. MW124301); and *L. ivanovii* strain Sr11 plasmid pLIS6 for *cadA6bC6b* (accession no. MW124302). The protein sequences encoded by the *cadA* and *cadC* genes were extracted from these GenBank files using custom Bash scripts: *cadA* and *cadC* for *cadA1C1; LMOh7858_pLM80_0083* and *LMOh7858_pLM80_0082* for *cadA2C2*; *lmo1100* and *lmo1102* for *cadA3C3*; *pLIS400101c* and *pLIS400106c* for *cadA6aC6a*; and *pLIS600081* and *pLIS600076* for *cadA6bC6b*.

The non-redundant CadA sequences were aligned with Clustal Omega (https://www.ebi.ac.uk/Tools/msa/clustalo/ (accessed on 18 March 2021)) the output of which was employed to identify the amino acids conserved only among those CadA proteins that confer resistance to a high concentration of cadmium (growth at 70 μg/mL) [41]. Also, this Clustal Omega alignment file was converted to FASTA format with Biopython and used to construct a phylogenetic tree with MEGA X using neighbor-joining method with 1000 bootstraps [42]. Conserved domains in CadA proteins were searched for with Batch-Conserved Domain Searches [39]. Non-redundant CadC proteins were analyzed in the same manner.

## 3. Results and Discussion

### 3.1. Isolation and Genome Sequencing of L. welshimeri Strains with Concurrent Resistance to Cadmium and Arsenic

Analysis of 66 samples from an urban surface water creek in Raleigh, NC, USA, for *Listeria* spp. revealed that three (4.5%) were positive for *L. monocytogenes* and 27 (40.9%) for other *Listeria* spp. Analysis of the isolates for resistance to cadmium and arsenic revealed that two of the samples yielded *Listeria* spp. with resistance to both cadmium (35 μg/mL, but not 70 μg/mL) and arsenic (500 and 1000 μg/mL, but not 2000 μg/mL), suggesting that their maximum tolerance levels for cadmium and arsenic lie between 35 and 69 μg/mL and between 1000 and 1999 μg/mL, respectively. The colonies were non-hemolytic on blood agar and, when tested with the multiplex PCR scheme of Doumith et al., they yielded only the *prs* band typical for several non-pathogenic spp. in the genus *Listeria* [28]. One colony from each sample was chosen for WGS, and analysis of the whole genome sequence data indicated that both strains were *L. welshimeri*. The sequenced strains were designated *L. welshimeri* SKWL416 (derived from a swab of a rock submerged in the creek; 22 January 2019; water temperature, 4.5 °C) and *L. welshimeri* SKWL425 (derived from a swab of a rock submerged in the same creek; 29 January 2019; water temperature, 9 °C). These *L. welshimeri* strains are hereafter referred to as SKWL416 and SKWL425, respectively.

WGS analysis indicated that the strains were highly similar, and only 24 single nucleotide polymorphisms were detected between them. In silico MLST analysis revealed that both strains belonged to the novel sequence type (ST) 2339. RFPlasmid analysis revealed that both strains harbored plasmids and all the plasmid-borne contigs were excluded from the genomic comparison analyses, resulting in an estimated size of 2.89 Mb and 2.86 Mb for the chromosomal genomes for SKWL416 and SKWL425, respectively. These genomes exhibited GC content of 36.1%, which is somewhat lower than *L. monocytogenes* (38.0%), as also reported before for *L. welshimeri* SLCC5334 [43]. Genomic comparison of these strains with BLAST2 and Mauve revealed that their chromosomal genomes were highly similar except for one large insertion (41.3 kb) observed only in SKWL416 (Figure 1). This SKWL416-specific insertion was located in a single contig, and its location was confirmed with PCR (data not shown). Analysis with the prophage prediction program PHASTER indicated that this insertion in the SKWL416 genome corresponded to a prophage (data not shown).

### 3.2. LGI2-3, a Chromosomal Genomic Island Homologous to the LGI2 of L. monocytogenes, Is Harbored by L. welshimeri Strains SKWL416 and SKWL425

As discussed earlier, the cadmium and arsenic resistance genomic island LGI2 (approx. 35 kb) has been found to be integrated in various locations in the *L. monocytogenes* chromosome and shows a propensity for serotype 4b, especially the hypervirulent clones CC1 and CC2, but was not previously encountered in non-pathogenic *Listeria* spp. [21]. One of the special features of LGI2 was that it harbored unique alleles of the *cadAC* cassette, *cadA4C4*, which conferred lower tolerance to cadmium (MIC, 50 μg/mL) than other *cadAC* alleles such as *cadA1C1*-*cadA3C3*, *cadA5C5*, *cadA6aC6a* or *cadA6bC6b*, which typically confer higher tolerance to cadmium (MIC ≥ 140 μg/mL) [16,19,23]. The fact that SKWL416 and SKWL425 exhibited the resistance profile typical for LGI2 in *L. monocytogenes*, i.e., growth in the presence of cadmium at 35 μg/mL but not at 70 μg/mL, as well as arsenic (500 μg/mL), led us to hypothesize that they may harbor LGI2 or a related genomic island. 

Analysis with Genome Comparator revealed that SKWL416 and SKWL425 indeed harbored homologs of LGI2 genes. Novel alleles that were identical between the two strains were detected for each homolog, suggesting that these strains harbored a novel LGI2 variant. BLAST2 with the LGI2 of *L. monocytogenes* Scott A as query against the genomes of SKWL416 and SKWL425 identified the homologous regions in the *L. welshimeri* strains, and these regions and their flanking genes were compared with each other. These analyses confirmed that *L. welshimeri* strains SKWL416 and SKWL425 harbored an identical LGI2 variant of 37.5 kb within single contigs in both strains (Figure 1A,B). This variant is hereafter referred to as LGI2-3, to differentiate it from LGI2-1 (a variant of LGI2 previously reported in certain *L. monocytogenes* strains of CC1) and LGI2-2, another variant identified in the *L. monocytogenes* CC1 strain J3422 [21]. LGI2-3 was harbored by SKWL416 and SKWL425 at the same chromosomal locus, immediately downstream of a gene encoding a putative glutamine-fructose-6-phosphate aminotransferase (Figure 1A,B and Figure 2). LGI2-3 exhibited a GC content of 34.4%, somewhat lower than the genome average for the *L. welshimeri* strains (36.1%), suggesting that this island might have been acquired via horizontal gene transfer [43]. In *L. monocytogenes*, the GC content of LGI2 and LGI2-1 (both 34.4%) was also lower than the average for the chromosome (38.0%) [21].

Given that LGI2 and its variants were previously detected only among *L. monocytogenes* strains and not in non-pathogenic *Listeria* spp. [21], it is noteworthy that a novel LGI2 variant was harbored by the aquatic *L. welshimeri* strains characterized in the current study. This finding suggests the intriguing possibility of the transfer of this island between different *Listeria* spp. Nonetheless, the prevalence of this island seems to be low in the *L. welshimeri* population. BLASTn searches of LGI2-3 against 187 *L. welshimeri* genomes available in the NCBI database as of 12 March 2021 failed to identify any that harbored the entire island. Homologs to a few LGI2-3 genes were detected in only 11 *L. welshimeri* genomes, and none of the homologs (except for one homologous to a part of *arsA2*) included genes predicted to mediate heavy metal resistance. The infrequent detection of LGI2-3 harboring *L. welshimeri* may reflect the possibility that this island might have been only recently acquired by strains in this species. It is also conceivable that presence of LGI2-3 may be linked with surface-associated survival of *Listeria* in aquatic ecosystems. It is noteworthy that both strains were isolated from swabs of submerged rocks in an urban freshwater creek. 

Few reports are available on *L. welshimeri* from aquatic and other natural habitats [44,45,46,47]. Interestingly, a study of urban and rural watersheds in Nova Scotia, Canada reported that *L. welshimeri* was more common in urban watersheds, while *L. innocu*a predominated in rural ones [47]. However, heavy metal tolerance of *L. welshimeri* from these watersheds was not described in this or previous studies investigating *Listeria* spp. in aquatic environments [44,45,46,47]. A recent analysis of cadmium and arsenic resistance in 119 non-pathogenic *Listeria* spp. isolates from water and soil revealed that arsenic resistance was mediated by Tn*554*, while LGI2 was not detected among any of the isolates [19].

The insertion site of LGI2-3 immediately downstream of the gene encoding a glutamine-fructose-6-phosphate aminotransferase was not previously encountered as an insertion site of LGI2 or LGI2-1 [21]. Identification of this novel insertion site provides further evidence supporting the concept that LGI2 and its variants represent “floating islands” capable of inserting themselves into various chromosomal locations [21].

### 3.3. LGI2-3 Exhibited the Characteristics of Multiple LGI2 Variants and Harbored Two Novel Genes

Comparison of LGI2-3 with other known LGI2 variants such as those harbored by *L. monocytogenes* strains Scott A (LGI2), OLM 10 (LGI2-1) and J3422 (LGI2-2) revealed that LGI2-3 harbored a gene encoding cystathionine beta-lyase in the same position as LGI2-1 (Figure 2A). However, LGI2-3 was generally more similar to the prototype LGI2, as reflected by the higher overall similarity to homologous regions in LGI2 than to those in LGI2-1 (sequence identity of 92.6 vs. 82.9%) (Figure 2A). Another noticeable feature was the novel cadmium resistance cassette (*cadA7C7*) in LGI2-3 (Figure 2). Genes in this cassette will be further discussed in the next section.

Previous analyses showed that the two ORFs at the 3′ end of LGI2 downstream of the gene encoding the LPXTG protein were conserved between LGI2 and LGI2-1, but unrelated in the LGI2-2 of *L. monocytogenes* J3422, which was otherwise identical to LGI2 [21]. Interestingly, in this same region immediately downstream of the LPXTG protein gene the LGI2-3 of *L. welshimeri* SKWL416 and SKLW425 harbored two novel ORFs (*SKWL416_00618* and *SKWL416_00619*) unrelated to those in LGI2/LGI2-1 (Figure 2A) or LGI2-2 (Figure 2B). Of further interest, BLASTp of SKWL416_00618 and SKWL416_00619 revealed that highly conserved homologs were not present in any of the other *L. welshimeri* genomes in the NCBI database but were detected in other *Listeria* spp., such as *L. seeligeri* and *L. monocytogenes,* as well as in *Enterococcus hirae*. Since these ORFs and their homologs were annotated as hypothetical proteins, we searched for conserved domains using Batch-Conserved Domain Searches to acquire a glimpse into their functions. SKWL416_00618 harbored a domain in the DNA_S_dndD superfamily (accession no. cI25734), which is associated with the DNA sulfur modification protein DndD, while no conserved domains were detected in SKWL416_00619. Taken together, our data indicated that LGI2-3 can be classified into a novel, hybrid LGI2 variant that exhibits characteristics of different LGI2 variants and harbors genes that are often found in non-*L. welshimeri Listeria* spp. and *E. hirae*, providing further support for the mosaic nature of this LGI2 variant.

### 3.4. Cadmium Resistance Determinants from LGI2 and Its Variants, Including LGI2-3, Cluster Together and Harbor Common Conserved Domains

**Figure 3:**
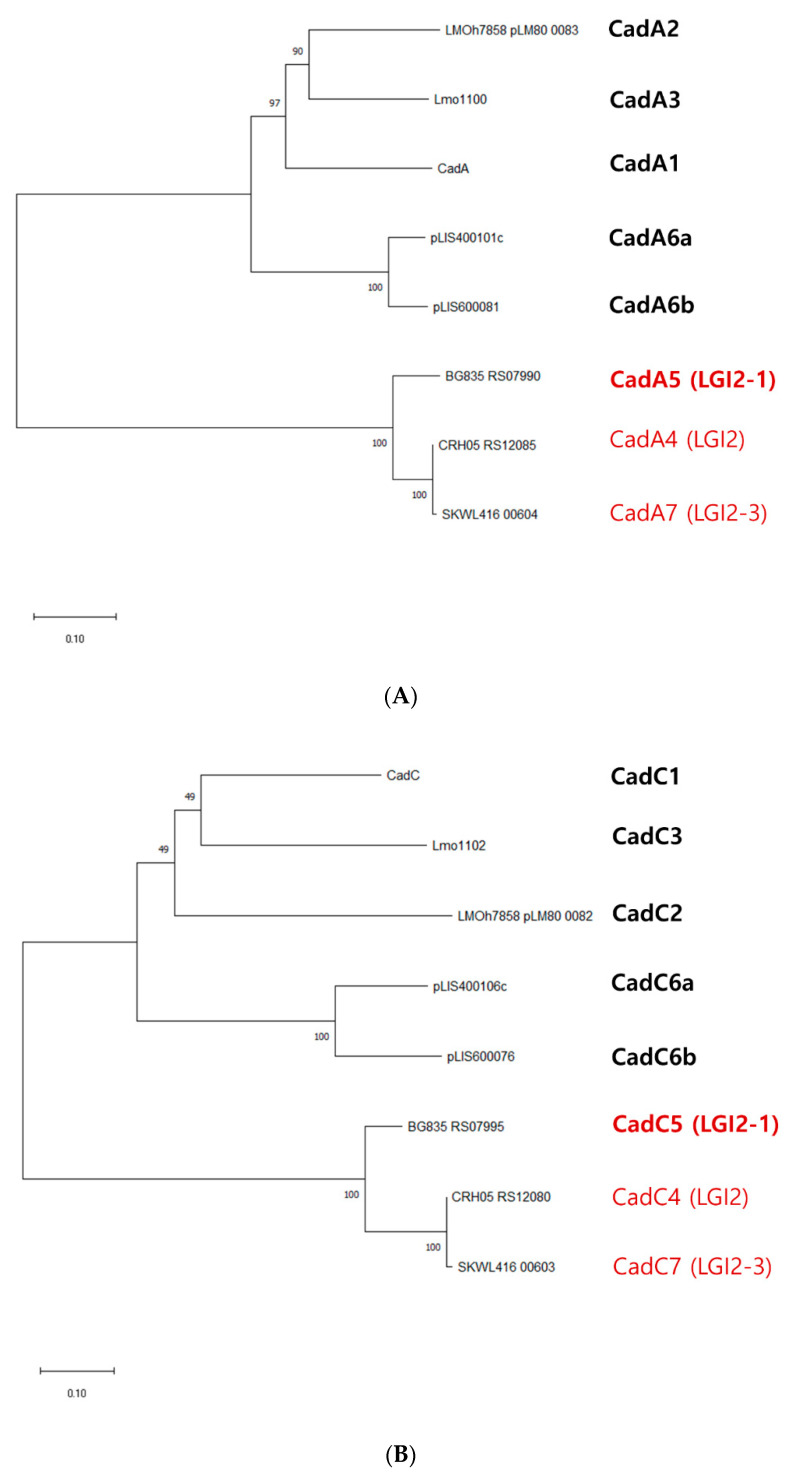
Phylogenetic trees of CadA (**A**) and CadC (**B**). The protein sequences for CadA and CadC were obtained as described in Materials and Methods from the GenBank files of the DNA sequences of Tn*5422*-associated *cadAC* (accession no. L28104) for CadA1 and CadC1; contig 506 of plasmid pLM80 harbored by *L. monocytogenes* H7858 (accession no. AADR01000058) for CadA2 and CadC2; *L. monocytogenes* EGD-e genome (accession no. NC_003210) for CadA3 and CadC3; LIG2 region extracted from *L. monocytogenes* Scott A genome (accession no. NZ_CP023862) for CadA4 and CadC4; LGI2-1 region extracted from *L. monocytogenes* OLM 10 genome (accession nos. NZ_MIMA01000001 to NZ_MIMA01000072) for CadA5 and CadC5; *L. seeligeri* Sr12 plasmid pLIS4 (accession no. MW124301) for CadA6a and CadC6a; *L. ivanovii* strain Sr11 plasmid pLIS6 (accession no. MW124302) for CadA6b and CadC6b; and *L. welshimeri* SKWL416 LGI2-3 (current study) for CadA7 and CadC7. Bootstrapping was generated with MEGA X as described in Materials and Methods [42]. The number at each branch represents the bootstrap value. Sequences associated with LGI2 and its variants are shown in red font and those conferring resistance to high concentrations of cadmium are marked in bold.

To date, multiple cadmium resistance determinants (*cadA1C1*-*cadA5C5*) have been identified in *L. monocytogenes*, with *cadA1C1* and *cadA2C2* also reported in *L. innocua* and *L. welshimeri* [16,17,18,19]. The most recent additions to this repertoire, *cadA6C6* in its two types a and b, were originally identified on plasmids of *L. seeligeri* and *L. ivanovii*, respectively [19]. With the exception of *cadA4C4* and *cadA7C7*, which are associated with growth at 35 μg/mL cadmium but not at 70 μg/mL (MIC, 50 μg/mL), the other cadmium resistance cassettes are associated with resistance to higher concentrations of cadmium, manifested by growth at 70 μg/mL and MIC ≥ 140 μg/mL [16,19,20]. Phylogenetic analysis of the different CadA protein sequences revealed that CadA7 was closest to CadA4, which, as discussed above, also conferred relatively low tolerance to cadmium (Figure 3A). However, the CadA proteins associated with LGI2 and its variants, i.e., CadA4, CadA5 and CadA7, clustered together in the phylogenetic tree, while the others formed a separate cluster (Figure 3A). Pairwise comparisons of CadA7 with other CadA proteins also showed that CadA7 was closest to CadA4 (99.4%) followed by CadA5 (89.6%) (Supplementary Material S3). In contrast, pairwise comparisons of CadA7 with non-LGI2-associated CadA proteins exhibited much lower similarities, ranging from 35.8 to 37.0% (Supplementary Material S3).

**Figure 4:**
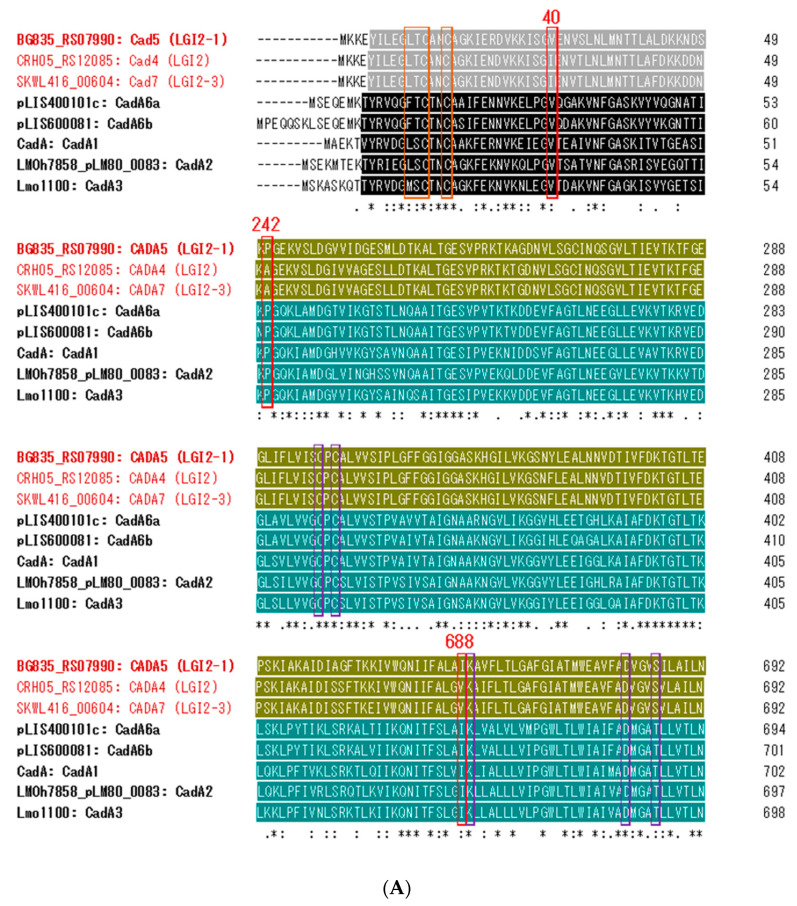

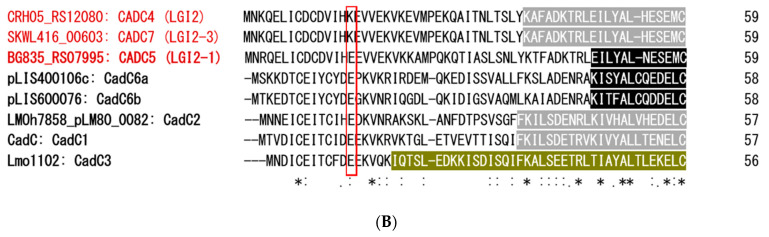
Amino acid sequence alignments of CadA and CadC proteins associated with different levels of tolerance to cadmium. Alignments of CadA (**A**) and CadC (**B**) protein sequences were generated via Clustal Omega [41]. Symbols “*”, “:” and “,” indicate the alignment of the identical, highly similar and dissimilar amino acids, respectively. Sequences associated with resistance to high concentrations of cadmium are in bold. Red font indicates sequences associated with LGI2 and LGI2 variants. CadA domains with accession nos. cd00371 and pfam00403 (superfamily cl00207) are in black and gray highlight, respectively, while those with accession nos. cd07545 and cd07548 (superfamily cl21460) are in teal and green, respectively. CadC domains with accession nos. cd00090, COG0640, and smart00418 (superfamily cl21459) are marked in gray, green, and black, respectively. Metal binding and putative heavy metal ion binding sites are marked with orange and purple rectangles, respectively. Amino acids that are conserved only among proteins with high-level resistance are in red rectangles and their locations are indicated. Location 688 of the CadA alignment is part of the putative heavy metal ion binding site.

The distinction between the LGI2-associated CadA proteins and the others is also reflected on the related but distinct conserved domains in the two clusters. CadA proteins associated with LGI2 and its variants harbored domains with accession nos. cd00371 and cd07545, whereas the counterparts in the other proteins have accession nos. pfam00403 and cd07548 (Figure 4A). The domains in the same regions belonged to the same superfamily: superfamily cl00207 for cd00371 and pfam00403, and superfamily cl21460 for cd07545 and cd07548 (Figure 4A).

The deduced CadC sequences also clustered based on LGI2 associations in the phylogenetic tree, which was supported by the CadC pairwise comparisons (Figure 3B and Supplementary Material S3). Interestingly, however, the conserved domains in CadC were not strongly associated with whether CadC proteins originated from LGI2 and its variants (Figure 4B). For instance, the domain with accession no. smart00418 was identified in CadC5 (LGI2-1), CadC6a and CadC6b while the domain with accession no. cd00090 was encountered in CadC1, CadC2, CadC4 (LGI2 and LGI2-2) and CadC7 (LGI2-3) (Figure 4B).

### 3.5. Two Amino Acids in CadA Might Be Important in Modulating the Tolerance Level to Cadmium

Previously we had noted that the metal binding motif CXXC differed between the low-tolerance determinant CadA4 (CANC) and the high-tolerance determinants CadA1, CadA2 or CadA3 (CTNC), and employed site-directed mutagenesis to examine the impact of the difference in levels of tolerance [23]. However, alteration of CANC on CadA4 to CTNC failed to confer increased tolerance to cadmium [23]. Further analysis of additional CadA sequences such as CadA5-CadA7 revealed that the CANC motif is also harbored by CadA5, which has high tolerance to cadmium (Figure 4A), providing further support that the different residue in this motif is not responsible for the differences in tolerance levels.

To identify amino acids that may be pivotal to determining resistance levels, we searched the CadA and CadC sequences for amino acids conserved only among those associated with high levels of resistance. Three amino acids at positions 40, 242 and 688, all within conserved domains, exhibited such characteristics: all the high-level resistance CadA proteins (CadA1-3, CadA5, CadA6a and Cad6b) possessed valine, proline and isoleucine, respectively, at these positions, while the corresponding locations were occupied by isoleucine, alanine and valine, respectively, in the low-level resistance CadA proteins CadA4 and CadA7 (Figure 4A). Given the highly similar characteristics of isoleucine and valine, the substitutions at positions 40 and 688 may be phenotypically silent. However, the residue at position 688 is part of a putative heavy metal ion binding site (Figure 4A) and the substitution at this motif merits further investigation. Meanwhile, the changes at position 242 involve the dissimilar amino acids, proline and alanine, and represent promising targets for future investigations into their potential role in determining the cadmium resistance level (Figure 4A).

In CadC, one amino acid at position 5 was conserved only among high-level tolerance CadC proteins, which bore glutamic acid at this location, whereas their low-level tolerance counterparts possessed lysine (Figure 4A). Even though these amino acids are highly similar and located outside the conserved CadC domains, their potential roles in determining tolerance levels is worthy of further investigation. Collectively, the findings can guide further experimental studies to investigate the CadA and CadC protein features important for mediating the different cadmium tolerance levels in *L. monocytogenes* and other *Listeria* spp.

## 4. Conclusions

We obtained clear evidence of the presence of a LGI2-like chromosomal island, LGI2-3, in two *L. welshimeri* strains recently isolated from an urban aquatic ecosystem in North Carolina, USA. As also observed with LGI2-harboring *L. monocytogenes*, the *L. welshimeri* strains with LGI2-3 exhibited concurrent resistance to cadmium and arsenic, with characteristic cadmium tolerance levels (growth at 35 μg/mL but not at 70 μg/mL). The detection and characterization of LGI2-3 in these *L. welshimeri* strains clearly indicates that non-pathogenic *Listeria* spp. can indeed serve as potential reservoirs for this island, which until now had only been noted in *L. monocytogenes*, primarily in certain hypervirulent clones of serotype 4b. Further studies are needed to characterize the potential of LGI2-3 to disseminate to other listeriae, and to elucidate its potential roles in the adaptive physiology and ecology of the bacteria, especially in aquatic ecosystems. 

## Data Availability

Not applicable.

## Supplementary Materials

The following are available online at https://www.mdpi.com/article/10.3390/biom11040560/s1, Supplementary Material S1: json file for the circular map of SKWL416, Supplementary Material S2: json file for the circular map of SKWL425, Supplementary Material S3: percent identity matrix generated by Clustal Omega with CadA and CadC proteins. All authors have read and agreed to the published version of the manuscript.
