# Supplementary material for: Identification and Characterization of a Novel Genomic Island Harboring Cadmium and Arsenic Resistance Genes in Listeria welshimeri
Source: Biomolecules. 2021 Apr 11;11(4):560. doi: 10.3390/biom11040560 (PMC8070118; doi:10.3390/biom11040560)
Supplement: Supplementary file 1 [file biomolecules-11-00560-s001.zip › Supplementary Material S3.docx]

CadA5 CadA4 CadA7 CadA6a CadA6b CadA1 CadA2 CadA3

1: BG835_RS07990 (CadA5) 100.00 89.86 89.57 36.70 35.82 35.75 36.30 35.66

2: CRH05_RS12085 (CadA4) 89.86 100.00 99.43 37.13 35.96 35.89 36.88 35.95

3: SKWL416_00604 (CadA7) 89.57 99.43 100.00 36.99 35.82 35.75 36.73 35.81

4: pLIS400101c (CadA6a) 36.70 37.13 36.99 100.00 91.03 65.62 63.36 64.33

5: pLIS600081 (CadA6b) 35.82 35.96 35.82 91.03 100.00 64.91 64.28 65.09

6: CadA (CadA1) 35.75 35.89 35.75 65.62 64.91 100.00 68.66 70.88

7: LMOh7858_pLM80_0083 (CadA2) 36.30 36.88 36.73 63.36 64.28 68.66 100.00 73.62

8: Lmo1100 (CadA3) 35.66 35.95 35.81 64.33 65.09 70.88 73.62 100.00

B.

CadC4 CadC7 CadC5 CadC6a CadC6b CadC2 CadC1 CadC3

1: CRH05_RS12080 (CadC4) 100.00 99.15 84.75 32.76 31.90 28.95 34.78 35.45

2: SKWL416_00603 (CadC7) 99.15 100.00 84.75 31.90 31.03 28.95 34.78 35.45

3: BG835_RS07995 (cadC5) 84.75 84.75 100.00 35.34 33.62 31.58 37.39 35.45

4: pLIS400106c (CadC6a) 32.76 31.90 35.34 100.00 76.23 46.22 47.06 44.14

5: pLIS600076 (CadC6b) 31.90 31.03 33.62 76.23 100.00 47.06 45.38 43.24

6: LMOh7858_pLM80_0082 (CadC2) 28.95 28.95 31.58 46.22 47.06 100.00 52.54 48.18

7: CadC (CadC1) 34.78 34.78 37.39 47.06 45.38 52.54 100.00 57.66

8: Lmo1102 (CadC3) 35.45 35.45 35.45 44.14 43.24 48.18 57.66 100.00

**Supplementary Material S3.** Percent identity matrix generated by Clustal Omega with CadA (**A**) and CadC (**B**) proteins.
